# Spontaneous Expulsion in Placental Presentations

**Published:** 1856-10

**Authors:** 


### SPONTANEOUS EXPULSION AND ARTIFICIAL EXTRACTION OF THE PLACENTA BEFORE THE CHILD, IN PLACENTAL PRESENTATIONS.

SECTION I.—*Dangers of Placental Presentations: Opinions of Authors: Statistical Evidence of the Fatality of these Presentations.*

All obstetric authors seem to agree on this point, that there is no one complication in midwifery attended with more anxiety to the practitioner, and few, if any, with more real danger to the patient, than cases of unavoidable hemorrhage from presentation of the placenta.

"Placental presentations," says Dr. F. Ramsbotham, "are always fraught with extreme peril." "The attachment of the placenta," observes Dr. Collins, "to the mouth of the womb, is one of the most dangerous complications to be met with in midwifery." "There are few dangers," to quote the words of Dr. Edward Rigby, "connected with the practice of midwifery, which are more deservedly dreaded, and which are wont to come more unexpectedly, both to the patient as well as to the practitioner, than that species of hemorrhage which occurs in cases where the placenta is implanted, either centrally or partially, over the os uteri." "It is," says Dr. Dewees, "confessed on all hands, that no accident attendant on conception, is equally menacing, as unavoidable hemorrhage; and it also emphatically declares to the physician, that much depends on him, that it shall not be very often fatal. "It is one," he adds, "of those extraordinary cases, in which nature does less for the preservation of the individual than in almost any other." "That form of hemorrhage," remarks Madame Lachapelle, "which depends upon the implantation of the placenta upon the internal orifice of the uterus, is one of the most dangerous accidents to which pregnant women are exposed." "It is perhaps," long ago observed Deleurye, "of all labors, that in which the mother and the child run the greatest danger." Still earlier, Lamotte states, that "amongst all the accidents of child-birth, there is not any one more perilous (*il n'y en a point un plus perileux*) than that in which the after-birth presents before the child."

The actual results of practice fully bear out the observations which I have selected from the preceding authors upon the danger of unavoidable hemorrhage from placental presentation. My friend, Dr. Churchill, in his late excellent work upon the *Theory and Practice of Midwifery*, has collected from different sources the records of 174 cases of this complication. Amongst these 174 cases, 48 proved fatal to the mothers; or nearly one in every three of them died. I have attempted to make a still more extensive analysis of recorded cases of placenta prævia, or placental presentation, and the consequences of this complication as bearing on the life of the mother. The following table shows the results of the inquiry:—

*Table of Maternal Mortality in Placental Presentations.*

| Reporters. | No. of Cases. | Mothers Lost. |
|---|---|---|
| Mauriceau, - - - - - | 18 | 3 |
| Portal, - - - - - - | 14 | 1 |
| Giffard, - - - - - - | 19 | 5 |
| Smellie, - - - - - - | 18 | 7 |
| Rigby, - - - - - - | 42 | 10 |
| Clarke and Collins, - - | 16 | 4 |
| Busch, - - - - - - | 13 | 2 |
| Schweighauser, - - - | 65 | 16 |
| Lachapelle, - - - - | 21 | 10 |
| J. Ramsbotham, - - - | 129 | 41 |
| F. Ramsbotham, - - - | 189 | 49 |
| Lever, - - - - - - | 34 | 8 |
| Lee, - - - - - - - | 46 | 14 |
| Wilson, - - - - - - | 20 | 10 |
| Total, - - - | 654 | 180 |

From the preceding Table, it thus appears that out of 654 cases of placental presentations which are collected into it, the result was fatal to the mother in 180 instances, or, in other words, 1 *in every* 3 6–10 *of the mothers perished in connection with this complication.*

5

The dangers of placental presentations to the mother may appear stronger to some minds if I state them in other terms. Two of the most fatal epidemics of modern times, are yellow fever and Indian or malignant cholera. In the well-known yellow fever of Gibraltar of 1828, the mortality among those attacked was nearly 1 in 4 1–2. In 1832–33, about one in 3 1–2 of those affected in England with the epidemic cholera, died. Hence those mothers who are the subjects of placental presentations, are submitted to as great peril of life from this obstetric complication, as they would be if seized with yellow fever or malignant cholera. Further, the operation of lithotomy is generally regarded as one of the most formidable in surgery, and is calculated to be fatal in the proportion of 1 in every 6 or 8 subjected to it. The occurrence of placenta prævia is twice as dangerous and fatal as the operation of lithotomy—1 in every 3 perishing under the first, and 1 in every 6 or 8 perishing under the last.

Looking at these results, it will, we believe, be readily conceded, that any attempt—such as is the professed object of the present memoir—to diminish this fearful maternal mortality in placenta prævia, is entitled at least to the consideration of the obstetric profession, even should it fail to be so fortunate as to secure their concurrence and conviction.

SECTION II.—*Recognized Principles of Treatment:—1. Evacuation of the Liquor Amnii; and, 2. Delivery by Turning. Proposal of a Third Principle: The Complete Separation of the Placenta; Grounds for Proposing it: Illustrative Cases.*

Hitherto, two great principles of treatment—if we leave out the minor details of management—may be said to have been pursued by obstetric practitioners in the treatment of placental presentations. And the two modes of practice I allude to are supposed by many to be applicable to two different stages or degrees of the complication. They consist of the two following measures:—

1. THE EVACUATION OF THE LIQUOR AMNII.

In some cases of placenta prævia, and under some circumstances, the artificial evacuation of the liquor amnii is recommended to be had recourse to, and thus the sam treatment is followed for "unavoidable" hemorrhage, as is followed by most practitioners in instances of "accidental" hemorrhage. This mode of practice has been especially applied of late to cases in which the presentation of the placenta was only *partial*, and where, consequently, a portion of the membranes was within reach, and to instances in which the hemorrhage was comparatively slight in its degree and effects. About a century and a half ago, the same treatment seems to have been employed also by some practitioners, in instances in which the placenta presented completely over the os uteri, the placental structure being perforated artificially by the finger or an instrument, in order to permit the liquor amnii to escape. After recommending that, in placenta prævia, the membranes should be pierced, or the fingers thrust through the placenta, "that at last it be perforated, and instead of the constant flux of blood which appeared before, the humors will presently flow out," Davanter, writing about the year 1700, adds, "some penetrate the secundines with a *hair needle*, which I do not approve of if it can be done with the fingers, because the infant is easily hurt." Under some conditions in placenta prævia, Deleurye recommends the piercing of the placenta with a trocar, in order to allow the liquor amnii to be drained off. Baudelocque speaks of the same practice as probably useful in instances of complete or central presentation of the placenta, when the cervix will not allow of turning; and in later years the same plan has been again proposed by the elder Dr. Ramsbotham, and successfully put in practice by Gendrin of Paris.

2. THE DELIVERY OF THE CHILD BY TURNING.

In the generality of cases of unavoidable hemorrhage from placental presentation, the practice which is adopted consists in forcing the delivery, by passing the hand through the os uteri up to the feet of the infant, and extracting the child by the operation of podalic turning. This last mode of practice is the one universally followed when the hemorrhage is very severe, and whether the artificial evacuation of the liquor amnii has preceded it or not, and it is the plan of treatment usually pursued where the presentation of the placenta over the os uteri is central or entire. By some accoucheurs indeed, as Drs. Burns and Hamilton, Baudelocque, Capuron, and others, the forcible delivery of the woman by the operation of turning is the *only* mode of treatment that is thought advisable under any circumstances in connection with *placenta prævia*. It is, according to Plenck, "nullo remedio sed sola extractione fœtus curanda." "All the best practical writers are," says Dr. Merriman, "unanimous on this point, that the case of placenta adhering over the cervix uteri is not to be trusted to nature. In all cases of attachment of the placenta over the os uteri, it is incumbent upon the accoucheur to make up his mind to the operation of turning the child, and bringing it into the world by the feet." "This is a case," Dr. Conquest remarks, "in which we ought never to confide in the powers of nature, because expulsatory uterine efforts only augment the peril of the patient; and therefore the hand must be either bored through the substance, or, what is better, passed by the edge of the placenta, and the child turned." "It is completely established," to quote the words of Dr. Dewees, "that the only chance the woman has for life, is by a well-timed and well-conducted delivery, in every case of placental presentation." "When hemorrhage," says Dr. Denman, "from this cause (placental presentation) comes on, though all women without proper assistance would not die, none are free from danger till they are delivered. As there is a very doubtful chance of the delivery by pains of labor, and as experience has fully proved the frequent insufficiency of all other methods intended to suppress the hemorrhage, and how little reliance ought to be placed on them, though they are always to be tried; it is a practice established by high and multiplied authority, and sanctioned by success, to deliver women by art, in all cases of dangerous hemorrhage, without confiding in the resources of the constitution. This practice is no longer a matter of partial opinion, on the propriety of which we may think ourselves *at liberty* to debate; it has for near two centuries met the consent and approbation of every practitioner of judgment and reputation in this and many other countries. See Mauriceau and almost every succeeding writer."

Cases of placenta prævia not unfrequently occur in practice in which neither of the two preceding plans can be successfully adopted—where the artificial evacuation of the liquor amnii is insufficient to moderate the hemorrhage to a safe degree—and where forced delivery by turning is inapplicable or extremely dangerous if adopted. In these and other cases, I would beg to submit to my obstetric brethren an additional principle of treatment, viz:

### 3. THE COMPLETE SEPARATION, AND, IF NECESSARY, EXTRACTION OF THE PLACENTA BEFORE THE CHILD.

I shall first state the grounds on which I venture to found the propriety of this proposed addition to the treatment of the very anxious and very dangerous cases of which we speak.

Obstetric pathologists seem unanimous in the opinion that all the more formidable varieties of hemorrhage which occur from the uterus in the latter months of utero-gestation, or the earlier periods of labor, are attributable to the separation of the vascular connections between the placenta and the interior of the uterus, and the escape of blood from the vessels which are laid open in consequence of this separation.

Paradoxical as it may appear, there are sufficient grounds and facts for believing, that when the placenta is separated slightly and partially, the chance of fatal hemorrhage to the mother is greater than when the disunion of the organ is entire and complete. Various authors have detailed cases in which the death of the mother speedily took place, though the portion of the placenta separated from the uterus was exceedingly small. Thus Dr. Hamilton mentions that in several cases which had fallen under his observation, and where he was called too late to afford proper assistance, it was discovered that the fatal hemorrhage had proceeded from the separation of "a very small portion of the placenta." In one instance of fatal hemorrhage between the 7th and 8th month of utero-gestation, he found on dissection that "the area of the separated placenta was less than a square inch."

On the other hand, I believe I have collected a sufficient number of data to prove that, when the disjunction of the placenta from the uterus is *perfect* and *complete*, the degree of maternal hemorrhage that occurs is in general exceedingly slight and trifling, or it is altogether arrested. The details of a few cases may illustrate and impress the fact which I wish to point out.

Case I.—*Placenta expelled upwards of three hours before the child; no hemorrhage in the interval; child removed by decapitation and extraction.*—In 1840 I was requested by my friend, Dr. Graham Weir, to see a patient at about the 5th month of pregnancy, who had been attacked with very severe hemorrhage. It was her third or fourth pregnancy. After the flooding had continued for some time, the placenta was expelled. *From the time of its expulsion the hemorrhage ceased.* The shoulder and neck of the infant were presenting over the os uteri. The os uteri was so contracted and the whole organ so small, as to prevent the possibility of the introduction of the hand for the operation of turning. At my suggestion, Dr. Weir severed the neck of the infant. Its body was then easily extracted by pulling at the presenting arm; and its head was immediately afterwards expelled by the unassisted action of the uterus. From three to four hours elapsed between the protrusion of the placenta and the complete delivery of the woman, yet during that time she lost little or no blood, and her recovery was speedy and perfect.

Case II.—*Placenta expelled about two hours before the child; elbow of the child presenting.*—In 1841, I was requested by Dr. Lewins, of Leith, to visit a case of complicated unavoidable hemorrhage. I saw the patient shortly after 9 o'clock in the morning. Labor pains had come on about 4, and a considerable degree of hemorrhage had accompanied them. Shortly after 7 o'clock, Dr. Lewins, on visiting the patient, found the placenta expelled through the os uteri. When I saw the woman, nearly two hours afterwards, the placental mass was lying between her thighs, and attached to her by the umbilical cord. She was weak from the hemorrhage that had occurred previous to the expulsion of the placenta, but from the time, that organ had been extruded, *the flooding had almost entirely ceased.* I found the elbow of the child presenting; and as the os uteri was well dilated, it was easy to bring down a lower extremity, and terminate the labor. The patient recovered without a bad symptom.

Case III.—*Placenta expelled some minutes before the child; no intervening hemorrhage; child expelled by natural pains, and revived.*—For the details of this case I am indebted to Dr. Dewar of Dunfermline, and shall give the circumstances in his own graphic words. "Some blood," he says, "had been lost as nearly as we could calculate at what would have been the seventh and eighth menstrual periods, and several times between the eighth and ninth months, and that in spite of an entire cessation from all exercise. Labor took place at the full time, and, as was dreaded, was accompanied with severe hemorrhage from the beginning. When I saw her, about an hour after pain had begun, the orifice of the uterus was pretty well dilated, and a soft spongy mass, apparently the centre of the placenta, protruded from it. There was no time for interference, for almost instantly a strong pain forcibly expelled the whole of the placenta from the vagina. To my surprise the flooding ceased. Pains continued active, and the child was born in less than ten minutes. After a little time the infant revived, and the mother recovered well, though considerably exhausted."

Case IV.—*Great hemorrhage, and expulsion of the placenta under strong uterine action; child extracted by turning some hours afterwards.*—Mrs. H., during her second pregnancy, her first child having been premature, had a slight flooding about the seventh month. When in the eighth month, labor commenced early on the morning of the 18th of May, with slight pains, and sanguineous discharge. These continued more or less severely till the evening of the 19th, when, as Mrs. H., was resting upon her knees and elbows, an immense gush took place, along with an unusually strong pain. Immediately afterwards, on being laid down, the placenta was found protruding from the external parts. The attendant midwife immediately sent off to a distance of several miles for two medical gentlemen, who arrived about half-past one o'clock on the morning of the 20th. In the meantime, the hemorrhage was inconsiderable. The medical men attempted to turn, and deliver the child, but encountered great difficulties in doing so, the head having remained fixed in the pelvis for an hour or two after the body was born. The recovery was tedious. The patient, now one of the most respected and intelligent midwives in Edinburg, has had three children since the above period.

CASE V.—*Unavoidable hemorrhage terminated by the expulsion of the placenta; child allowed to be delivered by the natural pains.*—"About half-past six on the morning of April 29th, 1818, a messenger," says Dr. Ramsbotham, "arrived at my house,, sent by two medical gentlemen, with a note to this purport: "We are in attendance upon Mrs. H., whose situation is involved in great uncertainty, from a placental presentation; the bleeding is going on pretty actively, and we wish for your immediate opinion." On my arrival at the house of the lady, about eight, I was told by one of the gentlemen, "that since the note was sent off, some strong expulsive pains had come on, which had expelled the placenta through the external parts before the head of the child, and that it was lying upon the bed. That before this occurrence the hemorrhage had been violent, yet not to that extent as apparently to endanger the woman's life; but that since the appearance of the placenta the flooding had very much abated. During our conversation on this unusual occurrence, the gentleman more immediately in attendance, who, at my arrival, was in the bed room of his patient, came down stairs and reported, "that the head was presenting at the brim of the pelvis, with a hand down by its side; that there was no want of uterine action; that the flooding had ceased; and that his patient did not seem much exhausted. An appeal was now made to my opinion, as to the further management of the case, to which I replied, "that as the flooding, the most dangerous sympton, had abated, as the labor-pains continued active, and especially as the woman's strength kept up, there did not appear to be an immediate necessity for a recourse to any means for hastening delivery; watch your patient for a short time, and wait the result; if the flooding should return, or if any dangerous symptom make its appearance, let us know." In about half an hour after this interview, the gentleman returned with a cheerful countenance, and stated that the child was expelled without further loss of blood, and that his patient was promising to do extremely well." &c.

CASE VI.—*Placenta gradually coming down, during the labor, into the os uteri, and being at last expelled four hours before the child; with no intermediate hemorrhage.*—Mrs. C., in the eighth month of her fourth pregnancy, was taken in labor on Sunday evening, about nine o'clock. Mr. Chapman was called to her about twelve o'clock. He was informed that the membranes had been ruptured for some time. The os uteri was dilated to the size of a crown piece, and the head presenting, but still very high. The pains were very strong and regular. On a second examination, an edge of the placenta was discovered "beginning to protude through the os uteri," with a hemorrhage which was trifling, but increased upon the return of the pains, though still inconsiderable as not to be alarming. Mr. C. did not hence conceive himself justified in proceeding to immediate delivery. But as upon every return of pain the placenta became more and more protruded through the os uteri, without the head advancing, the advice of another practitioner was sought. Previous, however, to his arrival, the pains proved so strong that the os uteri became dilated, and the placenta was completely expelled through the os externum, about three o'clock on Monday morning, with very little hemorrhage. From this moment the pain entirely ceased. The other practitioner did not arrive till five o'clock. "There had not," to use Mr. Chapman's own words, "been the least hemorrhage since the expulsion of the placenta." It was now resolved to turn the child; but after two prolonged attempts the feet could not be seized, the uterus being spasmodically contracted in the longitudinal direction, and the circular fibres appearing to act without the consent of the longitudinal. "During the whole of this time the hemorrhage had not in the least increased." Twelve drops of the tincture of opium were now administered. In a very short time the patient became easy and comfortable; and in less than half an hour the natural pains returned, and speedily expelled the child, with the head and arm presenting. Nothing remarkable happened in the convalescence, except a trifling attack of phlegmasia dolens, an affection from which the patient had likewise suffered after her first labor.

CASE VII.—*Placental and shoulder presentation; placenta expelled; turning.*—The patient, in the sixth month of her fifteenth pregnancy, was attacked with hemorrhage, that was alarming in extent, but not so great in quantity as to produce syncope. "I saw her," Dr. Ramsbotham writes, "two hours after the first attack of flooding. The placenta was now lying completely in the vagina, and there was not the least hemorrhage. The membranes were ruptured. The shoulder of the child presented. The cervix uteri was unexpanded and rigid, and it was consequently impossible to get my whole hand into the uterine cavity, but I succeeded in reaching the ham of the infant, and was by this means enabled to turn and deliver." "An hour or more" elapsed between the complete detachment of the placenta and the birth of the child. It had been dead for some time. The mother recovered perfectly.

CASE VIII.—*Placental and arm presentation; placenta in the vagina, and without hemorrhage, for about eight hours before the child was born.*—In a woman who had completed the full time of pregnancy, Dr. Macaulay found the placenta expelled from the uterus, and lying in the vagina. She had been flooding previously, but it had ceased about eight o'clock on the morning of the 13th of February, 1816. The late distinguished Dr. Kellie of Leith, visited the patient with him. The woman peremptorily refused to allow Drs. Macaulay and Kellie to deliver her. About four oclock, P. M., the pains quickened, the placenta was expelled from the vagina, and about half an hour afterwards an anencephalous infant followed. The child was in every way well shaped, except as regarded the head. "Dr. Kellie told me," to quote the note which Dr. Macaulay made at the time, "that the head was the smallest he had ever seen, and remarked, that though it was an axiom in midwifery, that when the placenta was implanted over the os uteri, hemorrhage *must* continue till the uterus was emptied, *yet here it stopped as soon as the placenta came down.*"—*Sympson's Obstetrical Works.*

